# Age and Intrinsic Fitness Affect the Female Rotator Cuff Tendon Tissue

**DOI:** 10.3390/biomedicines10020509

**Published:** 2022-02-21

**Authors:** Manuela Thierbach, Estelle Heyne, Michael Schwarzer, Lauren G. Koch, Steven L. Britton, Britt Wildemann

**Affiliations:** 1 Experimental Trauma Surgery, Department of Trauma, Hand and Reconstructive Surgery, Jena University Hospital, Friedrich Schiller University Jena, 07747 Jena, Germany; manuela.thierbach@med.uni-jena.de; 2 Department of Cardiothoracic Surgery, Jena University Hospital, 07747 Jena, Germany; estelle.heyne@med.uni-jena.de (E.H.); michael.schwarzer@med.uni-jena.de (M.S.); 3 Department of Physiology and Pharmacology, The University of Toledo, Toledo, OH 43606, USA; lauren.koch2@utoledo.Edu; 4 Department of Molecular and Integrative Physiology, University of Michigan, Ann Arbor, MI 48109, USA; brittons@med.umich.edu

**Keywords:** tendon, high-capacity runners (HCR), low-capacity runners (LCR), rotator cuff, histology, gene expression, aging

## Abstract

The risk of the development of tendon disorders or ruptures increases with age, but it is unclear whether intrinsic fitness during lifetime might also affect tendon properties. To investigate this, a contrasting rat model of high-capacity runners (HCR with high intrinsic fitness) and low-capacity runners (LCR with low intrinsic fitness) was employed. Histological and molecular changes in rotator cuff (RC) tendons from 10 weeks old (young; HCR-10 and LCR-10) and 100 weeks old (old; HCR-100 and LCR-100) female rats were investigated. Age-dependent changes of RC tendons observed in HCR and LCR were increase of weight, decrease of tenocytes and RNA content, reduction of the wavy pattern of collagen and elastic fibers, repressed expression of *Col1a1*, *Eln*, *Postn*, *Tnmd*, *Tgfb3* and *Egr1* and reduction of the *Col1*:*Col3* and *Col1*:*Eln* ratio. The LCR rats showed less physical activity, increased body weight, signs of metabolic disease and a reduced life expectancy. Their RC tendons revealed increased weight (more than age-dependent) and enlargement of the tenocyte nuclei (consistent with degenerative tendons). Low intrinsic fitness led to repressed expression of a further nine genes (*Col3a1*, *Fbn1*, *Dcn*, *Tnc*, *Scx*, *Mkx*, *Bmp1*, *Tgfb1*, *Esr1*) as well as the rise of the *Col1*:*Col3* and *Col1:Eln* ratios (related to the lesser expression of *Col3a1* and *Eln*). The intrinsic fitness influences the female RC tendons at least as much as age. Lower intrinsic fitness accelerates aging of RC tendons and leads to further impairment; this could result in decreased healing potential and elasticity and increased stiffness.

## 1. Introduction

Tendons are elastic structures connecting bone and muscle and are essential for joint movement. Pathological alterations of tendon tissue are called tendinopathy and are associated with decreased tendon function and lead to pain and restricted mobility of the joints. Although the research on tendinopathies has increased within recent decades, the etiology is not fully understood. Tendinopathy of the rotator cuff (RC) is a common musculoskeletal disorder, and incidence increases with age. A systematic review showed that the prevalence of RC diseases in asymptomatic patients younger than 20 years is 7%, while it increase to 56% in patients 80 years and older [1]. This age-related increase in RC diseases was also found in overall prevalence and in symptomatic patients. Furthermore, treatment of tendinopathies is not fully satisfactory due to a high failure rate. Healing of rotator cuff tears is reported to have a failure rate of 13% and factors affecting the healing are for example age, diabetes, hypertension, tear size and fatty infiltration [2]. The analysis of human rotator cuff tissue showed a significant decrease in cell biological characteristics and the expression and synthesis of the extracellular matrix protein collagen 1 as well as an altered expression of matrix-degrading enzymes in patients over 65 years of age [3,4]. However, the availability of human tissue is limited, and patients often suffer from other comorbidities that might affect the tissue. To closely investigate tendons, animal models are frequently used. Models for rotator cuff repair have been established, ranging from large to small animals such as rats that show anatomical features comparable to the human anatomy [5]. Use of animal models has experimental advantages, such as the collection of larger amounts of dissected tissue and not only biopsy samples, determined age, and good characterization, without or with defined comorbidities. A very interesting and unique animal model is the high- and low-exercise-capacity rats [6]. Two distinct rat lines were selectively bred from a genetically heterogeneous rat population: high-capacity runners (HCR) and low-capacity runners (LCR). After several generations, the rat lines differ significantly in their intrinsic (non-trained) maximal treadmill-running capacity and overall fitness. The HCR are healthy rats with high intrinsic fitness, while the LCR are sickly rats with low intrinsic fitness and a significantly reduced life expectancy [7]; LCR rats die at a median age of 24 months, whereas the HCR rats have a median lifespan around 35 months. The LCR showed an increase of cardiovascular risk factors and the development of a metabolic syndrome after 11 generations of selection [8]. Whether RC tendon tissue is altered in these animals has not yet been investigated. Two studies from Dirks et al. investigated Achilles’ tendon tissue from HCR and found no alterations after uphill running (combined with intratendinous collagenase injection) regarding histopathological changes [9,10]. Analyzing the tissues from HCR and LCR rats allows insights into changes in tendon biology regarding age in combination with metabolic fitness in genetically unmodified animals. We hypothesize that tendons of the rotator cuff show changes on the histological and molecular level with respect to animal age and intrinsic fitness.

## 2. Materials and Methods

### 2.1. Animal Model

Female high- and low-capacity runners (HCR, LCR) from generations 39 and 41 were used (young: offspring from generation 41; old: offspring from generation 39). The rats were housed under standard conditions with a temperature-controlled environment and 12 h light/dark cycle. They had unlimited access to food (Normal Chow: V1534 with 9 kJ% fat, 24 kJ% protein and 67 kJ% carbohydrates) and drinking water. This animal model has been described in detail in a previous publication [7]. No exercise training was performed. To characterize the rats, body weight, tibia size and blood glucose values were investigated. The LCR compared to HCR and the old rats compared to young rats showed a significantly higher body weight. The tibia of old rats was significantly longer than that of young rats. In young HCR, the fasting glucose value was significantly lower than in young LCR and the glucose tolerance was significantly higher in old HCR than in old LCR.

Animals with an age of 10 and 100 weeks were sacrificed, and the rotator cuffs harvested for further histological and molecular analysis. In accordance with the 3R principles, the tissue was obtained from animals used in another experiment, which was approved by the local authorities (Thüringer Landesamt für Verbraucherschutz, permission number: 02-082/14, approval date: 14 November 2014). In this experiment, no treatment was performed that could have an impact on the tendon. In total, tissue from 28 rats was obtained. Animals were weighed and deep anesthesia was induced using thiopental (150 mg/kg bodyweight) before animals were sacrificed.

### 2.2. Sample Collection

The right and left rotator cuff from each rat was prepared. The left rotator cuffs (bone, tendons and muscle) were used for histological evaluation. The tissues were fixed in 4% paraformaldehyde and stored at 4 °C for 48 h. From the right rotator cuff the supraspinatus and infraspinatus tendons were carefully dissected and the musculature and surrounding tissue were eliminated. The tendons were used for gene expression analysis. RNA isolation was performed after snap-freezing in liquid nitrogen and storage at −80 °C. 

### 2.3. Histology

After washing in PBS, the left rotator cuffs were embedded in methyl methacrylate (Technovit 9100, Kulzer, Wehrheim, Germany). Thin sections (10 µm) were cut with a microtome (Polycut S, Reichert-Jung Optische Werke AG, Wien, Austria). For histological staining, sections were deplastinated in 2-Methoxyethylacetate and rehydrated in a descending alcohol series finishing off with ethanol 50%. Only sections showing the entire supraspinatus enthesis were stained. Hematoxylin Eosin (HE) staining was performed with Mayer’s hemalum solution (Merck KGaA, Darmstadt, Germany) and Eosin G 0.1% (Merck KGaA). For Movat Pentachrome staining (MP), sections were stained with alcian blue 1% (Merck KGaA) for 10 min at room temperature. Then, sections were washed in water for 5 min and treated with alkaline ethanol for 60 min. After 10 min washing, cell nuclei and elastic fibers were stained with Weigert’s Hematoxylin (Solution A and B 1:1, Waldeck GmbH & Co. KG, Münster, Germany) followed by the staining of the cytoplasm with Brilliant-Crocein-Acid Fuchsine (Waldeck) for 12 min. Sections were differentiated with acidic acid 0.5%, pickled with Phosphotungstic acid 5% (Carl Roth GmbH & Co. KG, Karlsruhe, Germany) for 20 min and again differentiated with acidic acid 0.5%. After dehydration with ethanol 100%, collagen fibers were stained with Safron du Gatinais (Waldeck) in 100% ethanol for 60 min.

### 2.4. Image Analysis

The HE stained sections were digitalized with a slide scanner (Hamamatsu Photonics Europe GmbH Herrsching, Germany) and viewed with the NDP.view 2.7.52 software (Hamamatsu Photonics). The analysis was performed with ImageJ 1.52p software (Wayne Rasband, National Institute of Health, Bethesda, MD, USA). Two representative fields of 40× magnification from the first third (initiating with the enthesis) of each supraspinatus tendon were chosen. To contrast the surrounding tissue, the tenocyte nuclei were selected manually with the freehand tool. The following parameters were analyzed: number, area, perimeter, circularity, minor and major dimension of the nuclei.

### 2.5. qRT-PCR

Tendon samples stored at −80 °C were homogenized with TRIzol (Thermo Fisher Scientific, Waltham, MA, USA) using a T25 digital ULTRA-TURRAX (IKA, Staufen, Germany). Phase separation was performed using chloroform. The RNA was purified with the RNeasy Plus Mini Kit (Qiagen, Hilden, Germany) according to the manufacturer’s instructions and afterwards quantified with the NanoDrop 2000c spectrophotometer (Thermo Fisher Scientific, Waltham, MA, USA). Exemplary analysis of RNA integrity was performed with the Agilent 2200 TapeStation system (Agilent technologies, Santa Clara, CA, USA) and revealed good RNA quality with only slight RNA degradation. cDNA synthesis of 100 ng RNA template was performed with the qScript cDNA Supermix (Quanta Bioscience, Gaithersburg, MD, USA). qRT-PCR was performed with the SYBR^®^ Green SuperMix (Quanta Biosciences) and a Rotor-Gene Q (Quiagen). All primer sequences except the scleraxis primer (ordered at Qiagen, Cat No.: QT01596028) were designed using Primer-3- and NetPrimer-software (PREMIER Biosoft International, San Francisco, CA, USA) and produced by TIB Molbiol, Berlin, Germany (sequences are shown in Table 1). The *18s rRNA* was chosen as housekeeping gene because it was most constant compared to hypoxanthine phosphoribosyl transferase (*Hprt*) and Glycerinaldehyd-3-phosphat-Dehydrogenase (*Gapdh*). All primers were tested for amplification efficiency and the ΔCt method with efficiency correction was used to calculate the relative gene expression to the reference gene 18S rRNA. The normalized expression was calculated using the following efficiency corrected equation according to Simon [11]:

$$\mathrm{NE}=\frac{{\left({\mathrm{Primer}\;\mathrm{efficiency}}_{\mathrm{Reference}}\right)}^{C_{q}{}_{\mathrm{Reference}}}}{{\left({\mathrm{Primer}\;\mathrm{efficiency}}_{\mathrm{Target}}\right)}^{C_{q}{}_{\mathrm{Target}}}}$$

In total, the expression of 18 genes was analyzed. For gene expression analysis seven samples per group (HCR-10, HCR-100, LCR-10, LCR-100) were investigated.

### 2.6. Statistics

The data are given in the text or shown in dot blots. The effect of aging within HCR and LCR, and the effect of fitness in age-matched groups was compared. For multiple group comparison of the non-parametric data the Kruskal–Wallis test followed by Mann–Whitney and Bonferroni–Holm correction was performed (SPSS 26, IBM, Armonk, NY, USA). A *p*-value of ≤0.05 was considered statistically significant.

## 3. Results

This study investigated the effect of age and intrinsic fitness on the histology and gene expression of rat rotator cuff tendons. Rats 10 weeks of age (HCR-10 and LCR-10) are comparable to young sexually mature adolescent humans, while rats 100 weeks of age (HCR-100 and LCR-100) correspond approximately to humans 60 years of age. However, this age correlation might be influenced by the rat strain and is not absolute. 

**Figure 1:**
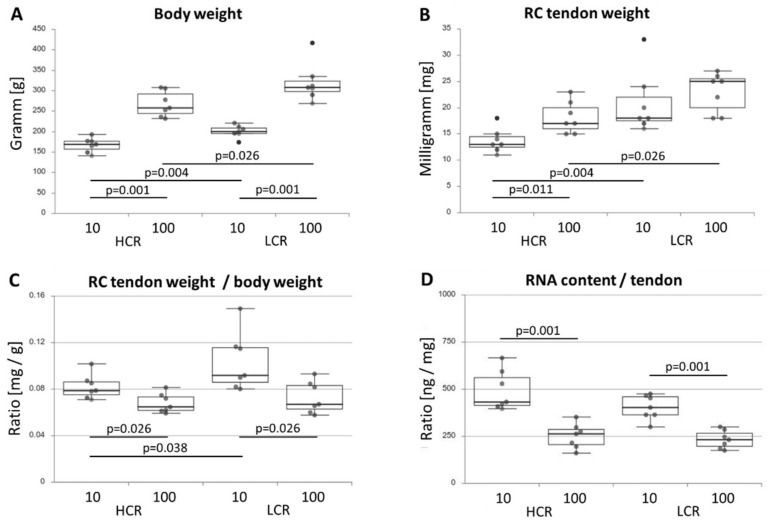
Body weight [g] (**A**), weight of the RC tendon [mg] (**B**), ratio of the RC tendon weight to body weight [mg/g] (**C**), and RNA content per tendon [ng/mg] (**D**) from HCR-10, HCR-100, LCR-10 and LCR-100. Significant differences (analyzed using the Kruskal–Wallis test followed by the Mann–Whitney U-test) are marked and the *p*-value displayed above. n = 7 per group.

Body weight was significantly higher in the less fit LCR rats compared to HCR rats and in old rats compared to young rats independent of the intrinsic fitness (Figure 1A). The RC tendon weight was also significantly different, with the lowest weight in HCR-10 and the highest in LCR-100 (Figure 1B). Calculating the ratio of the RC tendon weight to body weight (Figure 1C), LCR-10 had the most massive RC tendons in relation to body weight. In old rats, the RC tendon-weight-to-body-weight ratio was smaller compared to young rats due to increasing body weight with age. The RNA content per tendon was significantly lower in old compared to young rats (Figure 1D).

### 3.1. Histology of the Rotator Cuff

**Figure 2:**
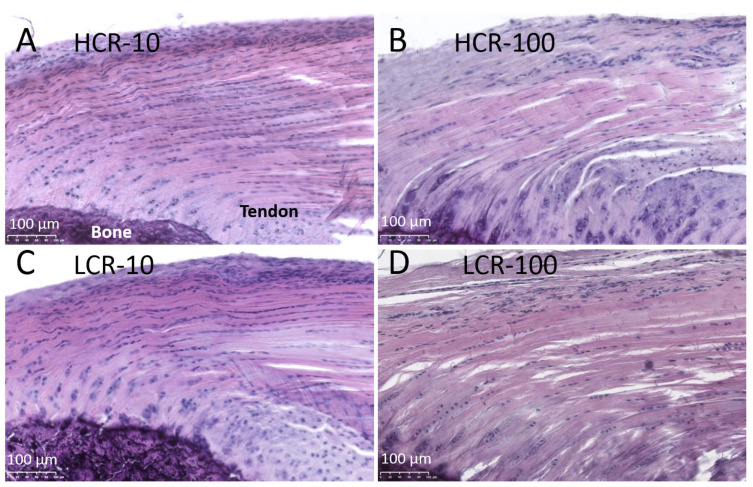
Histology of supraspinatus tendons from HCR-10 (**A**), HCR-100 (**B**), LCR-10 (**C**) and LCR-100 (**D**) using Hematoxylin Eosin (HE) staining. The tendons of the young rats showed a dense and aligned collagen structure with high cellularity. In the older rats, the structure was less dense with reduced number of cells. Scale bar: 100 µm. As marked in (**A**), the bone is always at the lower left corner.

The histological analysis of the first third (initiating with the enthesis) of the supraspinatus tendon showed intact supraspinatus tendons with collagen fiber bundles arranged in direction of mechanical load (Figure 2). In young HCR (Figure 2A) and LCR (Figure 2C) the supraspinatus tendon had a tightly packed structure. In the old rats, the tendon was less dense, and a decreased collagen matrix organization was observed, which was more pronounced in LCR-100 (Figure 2D) than in HCR-100 (Figure 2B). 

The tenocytes were arranged in rows with longer rows in the young than in the old rats. In young LCR rats, the tenocytes nuclei were slightly rounder and larger than in the young HCR rats, whereas the old LCR rats showed significantly (*p* = 0.004) larger tenocyte nuclei than the old HCR rats (Table 2).

**Figure 3:**
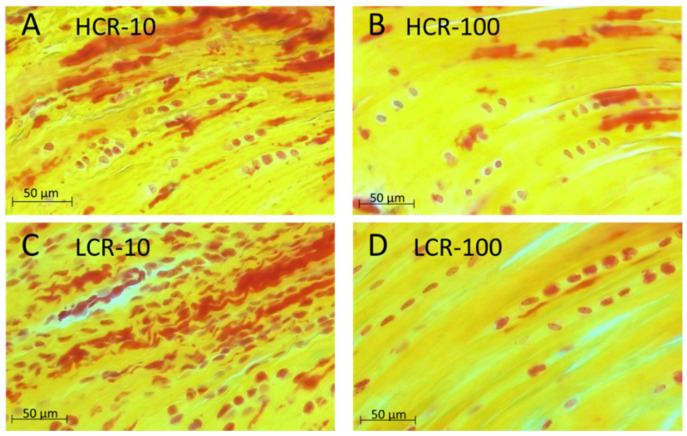
Histological staining of supraspinatus tendons of HCR-10 (**A**), HCR-100 (**B**), LCR-10 (**C**) and LCR-100 (**D**) using Movat Pentachrome (MP) staining. The number of elastic fibers and the wavy structure of the tendons of the young rats were reduced in the old tendons. Cell nuclei were stained in red; collagen in yellow and elastic fibers in red. Scale bar: 50 µm. The bone would be on the left side and the muscle on the right side of the tendon samples shown.

Using Movat Pentachrome staining (Figure 3) the collagens were stained yellow and the elastic fibers red. Elastic fibers support the recovery of the collagen wave-like pattern after stretching. The elastic fibers were distributed within tendons. The number of elastic fibers was higher in the young than in the old tendons, and the elastic fibers in the young tendons were wavier, similar to collagen fibers.

### 3.2. Molecular Analysis of the Rotator Cuff Tendons

**Figure 4:**
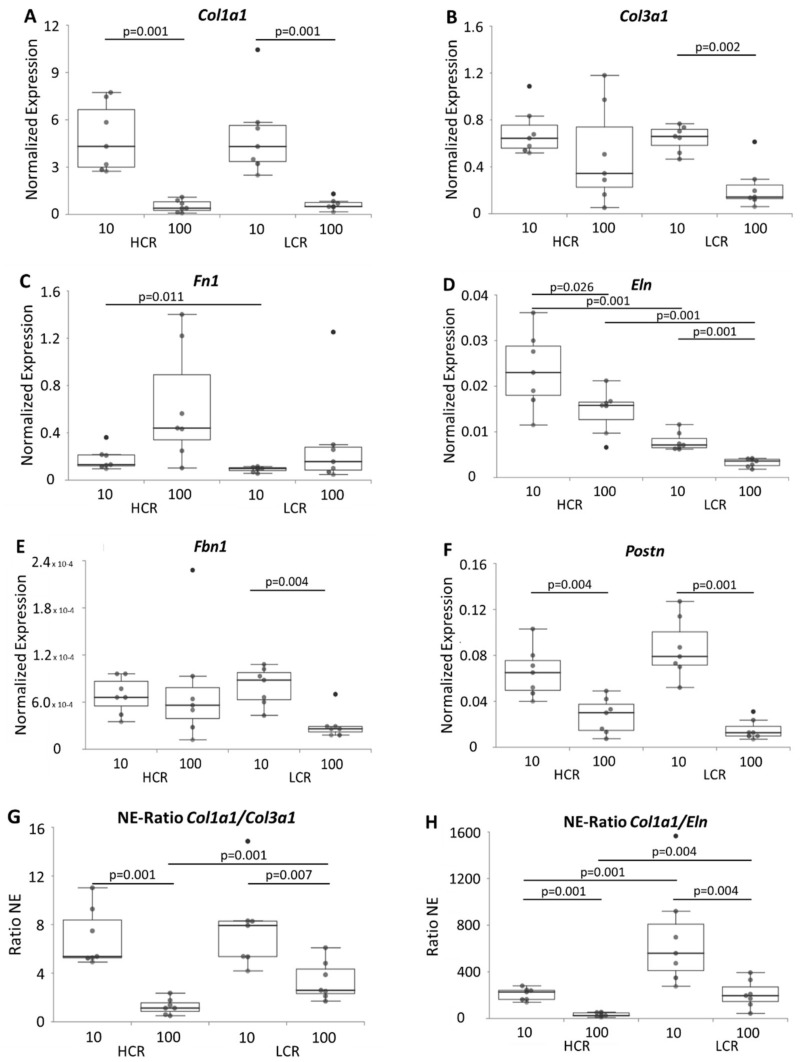
mRNA expression of ECM markers *Col1a1* (**A**), *Col3a1* (**B**), *Fn1* (**C**), *Eln* (**D**), *Fbn1* (**E**) and *Postn* (**F**) and Ratio of the NE *Col1a1*:*Col3a1* (**G**) as well as *Col1a1:Eln* (**H**) in HCR-10, HCR-100, LCR-10 and LCR-100. Results are normalized to 18S rRNA and are shown as individual dot plots. Significant differences (analyzed using the Kruskal–Wallis test followed by the Mann–Whitney U-test and Bonferroni–Holm correction) are marked and the *p*-value displayed above. n = 7 per group.

**Figure 5:**
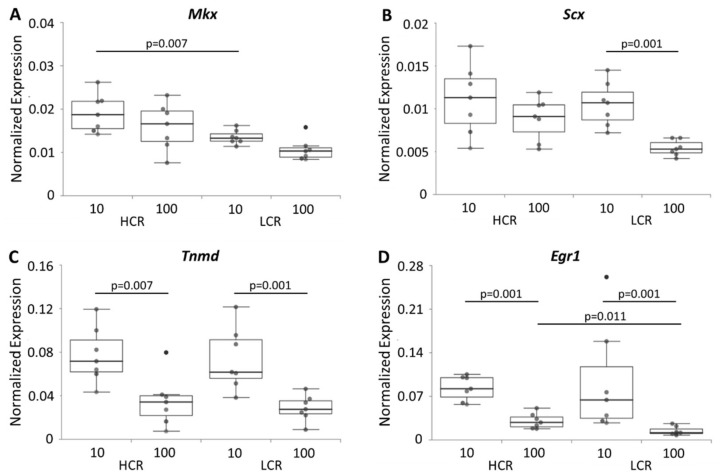
mRNA expression of the tenocyte markers *Mkx* (**A**), *Scx* (**B**), *Tnmd* (**C**) and the transcription factor *Egr1* (**D**). Results are normalized to 18S rRNA and are shown as individual dot plots. Significant differences (analyzed using the Kruskal–Wallis test followed by the Mann–Whitney U-test and Bonferroni–Holm correction) are marked and the *p*-value displayed above. n = 7 per group.

**Figure 6:**
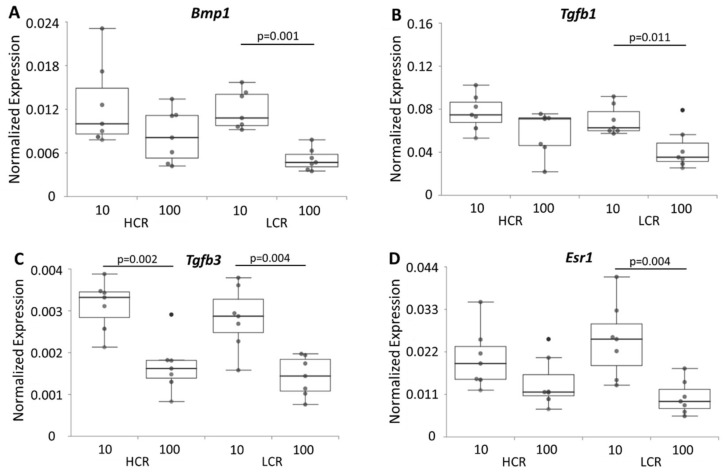
mRNA expression of the metalloprotease *Bmp1* (**A**), the cytokines *Tgfb1* (**B**), *Tgfb3* (**C**) and the sex hormone receptor *Esr1* (**D**). Results are normalized to 18S rRNA and are shown as individual dot plots. Significant differences (analyzed using the Kruskal–Wallis test followed by the Mann–Whitney U-test and Bonferroni–Holm correction) are marked and the *p*-value displayed above. n = 7 per group.

The expression of several extracellular matrix genes, tendon-specific genes, transcription factors and growth factors is important for the maintenance and function of the tendon, and might be influenced by intrinsic fitness and age. The expression of genes important for formation and modelling of the extracellular matrix such as collagen 1a1 (*Col1a1*), collagen 3a1 (*Col3a1*), decorin (*Dcn*), biglycan (*Bgn*), periostin (*Postn)*, elastin (*Eln*), fibrillin 1 (*Fbn1*) and fibronectin (*Fn*) was investigated. In addition, the tenocyte markers tenomodulin (*Tnmd*), mohawk (*Mkx*), tenascin-C (*Tnc*), scleraxis (*Scx*), the transcription factor early growth response 1 (*Egr1*), the cytokines transforming growth factor beta 1 (*Tgfb1*) and 3 (*Tgfb3*), the protease bone morphogenetic protein 1 (*Bmp1*) that cleaves the C-terminus of procollagen I, II and III, and the sex hormone receptors estrogen receptor alpha (*Esr1*) and androgen receptor (*Ar*) were analyzed. In the tendons of aged LCR, 12 genes were down-regulated, while in aged HCR only six genes were expressed lower in comparison to the young tendons (Figure 4, Figure 5 and Figure 6). In young vs. old HCR these genes were *Col1a1*, *Eln*, *Postn* (Figure 4A,D,F), *Tnmd*, *Egr1* (Figure 5C,D) and *Tgfb3* (Figure 6C); in young vs. old LCR additionally down-regulated genes included *Col3a1*, *Fbn1* (Figure 4B,E), *Scx* (Figure 5B), *Bmp1*, *Tgfb1* and *Esr1* (Figure 6A,B,D). Comparing HCR and LCR of the same age, the expression of only a few genes (3 genes at 10 weeks; 2 genes at 100 weeks) was significantly changed. In the tendons of 10 weeks old rats these were *Fn1*, *Eln* (Figure 4C,D) and *Mkx* (Figure 5A) while in the 100 weeks tendons these were *Eln* (Figure 4D) and *Egr1* (Figure 5D). All genes were down-regulated in LCR compared to HCR. These data reveal that the age as well as the intrinsic fitness had an impact on the regulation of the expression of genes. This fact is seen very pronounced by the expression of *Eln* (Figure 4D). It was changed in all four comparisons (HCR-10/100, LCR-10/100, 10-HCR/LCR, 100-HCR/LCR), which means in age as well as intrinsic fitness-dependent manner. The expression of *Dcn*, *Bgn*, *Tnc* and *Ar* was not influenced neither by age (when comparing HCR-10/100 and LCR-10/100) nor by intrinsic fitness (when comparing 10-HCR/LCR and 100-HCR/LCR).

The ratio of *Col1*:*Col3* was significantly reduced by age, but increased in LCR-100 compared to HCR-100 (Figure 4G). The *Col1*:*Eln* ratio was also affected with a significant change due to age and higher intrinsic fitness (Figure 4H).

**Figure 7:**
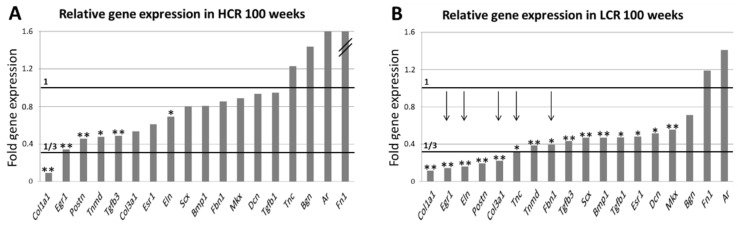
Effect of intrinsic fitness on gene expression. Relative gene expression of 18 genes in HCR-100 (**A**) and in LCR-100 (**B**) given as fold change to HCR-10 (horizontal line, mean value = 1). Significant differences (Mann–Whitney U-test) between HCR-10 and HCR-100 and between HCR-10 and LCR-100 are marked with * (*p* ≤ 0.05) or ** (*p* ≤ 0.005). The vertical arrows in (**B**) mark reduction in gene expression of ≥60% in comparison to (**A**).

In the next step, a special focus was set on the effect of low intrinsic fitness. To achieve this, HCR-10 were defined as fit rats and the influence of age (Figure 7A, relative gene expression in HCR-100) was compared with the combined influence of age and low intrinsic fitness (Figure 7B, relative gene expression in LCR-100). In the tendons of old HCR (influence of age; Figure 7A) the expression of six genes was significantly reduced; in old LCR (influence of age plus low intrinsic fitness; Figure 7B) 15 genes were significantly reduced. This means age resulted in a down-regulation of gene expression while age paired with low intrinsic fitness resulted in a stronger down-regulation of gene expression. In HCR-100 tendons (Figure 7A) the expression of *Col1a1* was lower than one third (shown as horizontal 1/3 line) of the HCR-10 gene expression. In LCR-100 tendons (Figure 7B) the expression of *Col1a1*, *Egr1*, *Eln*, *Postn*, *Col3a1* and *Tnc* showed such a strong reduction. In addition, especially *Egr1*, *Eln*, *Col3a1*, *Tnc* and *Fbn-1* were strongly down-regulated in comparison to HCR-100; the vertical arrows mark reduction of gene expression for ≥60%.

## 4. Discussion

Tendinopathy seems to be a multifactorial process significantly influenced by age and health status. The present study aimed at investigating the histological and molecular characteristics of the RC tendons of young vs. old as well as fit HCR vs. less fit LCR rats. Histological and molecular alteration were analyzed and compared between HCR and LCR to better understand possible reasons for the development of degenerative tendon pathologies. Our results indicated that age as well as fitness influenced the female RC tendons strongly.

### 4.1. The Effect of Age on the Rat Rotator Cuff Tendon

Old rats, independent of their intrinsic fitness, presented with significantly higher body weight and RC tendon weight than the young rats. The histological investigations also showed differences in the supraspinatus tendons between young and old rats. More than twice as many tenocytes were counted in tendons of young rats compared to old. Tenocytes are the major cell type in tendons and the reduction of the cell number with age was also reported by Ippolito et al. [12] for rabbit Achilles’ tendons and by Dunkman et al. [13] for mice patellar tendons. The decreased cell number (about two-fold) could explain the significant reduction of the RNA content per tendon in the old rats (also about two-fold). Furthermore, the tendons of young rats exhibited a wavy or crimped pattern of collagen and elastic fibers, which was reduced in the old rats. The structural properties may play an important role in tendon elasticity and response to fatigue loading [14]. This might indicate that the supraspinatus tendons of old rats were less elastic and stiffer compared to young rats. 

Additionally, there was an age-related repression of gene expression in both HCR and LCR. In young vs. old RC tendons *Col1a1*, *Eln*, *Postn*, *Tnmd*, *Tgfb3* and *Egr1* were down-regulated. This can be seen as an effect of normal (natural) aging. Collagen type I is the major structural component of the tendon, and its age-dependent repression was also reported for rat [15], horse [16] and for human tendons [17]. ELN is the core protein of elastic fibers and ensures elastic stretching and recoiling of tissue, cooperating with collagen for tensile resistance [18]. For horse tendons, Godinho et al. [19] reported an age-dependent ELN reduction. The age-dependent repression of *Col1a1* and *Eln* was confirmed with the histological result showing a loss of crimped pattern of collagen and elastic fibers in the supraspinatus tendon of old rats. POSTN is a secreted ECM protein involved in the regulation of cell–cell and cell–matrix interactions [20] and promotes tendon regeneration. It interacts with collagen type I and plays an important role in collagen fibrillogenesis [21]. In accordance with our results, Wang et al. [22] found that *Postn* was expressed at high levels in early rat tendon development and was reduced in mature tendons. TNMD is a tendon-specific marker known to be important for tendon maturation with key implications for the residing tendon stem/progenitor cells. An age-dependent down-regulation of *Tnmd* was described in horse tendons [16] but also in mice intervertebral discs and has been postulated as a risk factor for age-related degeneration [23]. Regarding the healing of rotator cuff tears it has already been shown that the sustained delivery of TGFB3 accelerated the healing process [24] and in mouse Achilles’ tendons canonical TGFβ signaling has a functional role in tendon regeneration [25]. EGR1 is a transcription factor and important for embryonic and postnatal tendon development [26]. It induces tenogenic differentiation of tendon stem cells and promotes rabbit rotator cuff repair [27]. *Egr1* is regulated by mechanical loading and a lack of EGR1 is associated with weaker tendon formation [28]. Thus, age influences the expression of various genes important for formation, maturation, maintenance and regeneration of the tendon extracellular matrix.

The ratio of *Col1*:*Col3* and of *Col1*:*Eln* was significantly reduced by age in HCR as well as in LCR tendons. This was caused by the strong age-dependent down-regulation of the *Col1a1* expression, which was more pronounced than the down-regulation of *Col3a1* and *Eln*, respectively. Collagen type I is the predominant collagen type in tendon tissues, followed by collagen type III. Alterations affect the formation of collagen fibrils and the ECM, which globally impairs tendon structure, biomechanics and tendon repair and may contribute to pathogenesis of tendinopathy. Change of the ratio of *Col1a1*:*Eln* has been considered to be a sign of developing lumbar spinal stenosis in elder patients [29] and the COL:ELN ratio was described as an accurate predictor of arterial burst pressure [30]. 

The repression of the expression of the investigated genes and the histological changes described above may be associated with increased risk of tendon injury with aging.

### 4.2. The Effect of Low Intrinsic Fitness on the Rat Rotator Cuff Tendon

HCR and LCR rats are a well-characterized animal model, showing significant differences in the intrinsic running capacity/fitness [7]. The low intrinsic fitness associated changes of the LCR tendons, which were found in the present study, may be explained by several comorbidities associated with this model. The body weight of the LCR-10 and LCR-100 was significantly higher compared to the HCR-10 and HCR-100, indicating an obese phenotype of LCR, which is a clinical picture of the metabolic syndrome. The RC weight was significantly higher in LCR-10 and LCR-100 than in HCR-10 and HCR-100. Correlating tendon weight to body weight, LCR-10 showed higher values than HCR-10 and LCR-100. Taking these data together, LCR developed massive RC tendons early in life, but not HCR. This increase in tendon thickness is in line with human data. Akturk et al. [31] reported that the supraspinatus tendons in diabetic patients become thickened. In a diabetic rat model, it was observed that the thickness of the Achilles’ tendons was significantly increased in the diabetic animals in comparison to the control group [32].

The histological investigations showed an increased nuclear size and rounding of the nuclei especially in old LCR, which is consistent with degenerative tendons and was also observed in diabetic mouse Achilles’ tendons [33]. Furthermore, the observed increased interfibrillar spaces were also reported for Achilles’ tendons of diabetic patients [34]. All these results supported differences between HCR and LCR RC tendons and were confirmed by molecular analysis. To differentiate the pure effect of low intrinsic fitness, the gene expression in RC tendons of HCR-10 vs. HCR-100 was compared with HCR-10 vs. LCR-100. In HCR-10 vs. HCR-100 *Col1a1*, *Eln*, *Postn*, *Tnmd*, *Tgfb3* and *Egr1* were down-regulated and this was defined as an effect of normal aging (see Section 4.1 above).

In HCR-10 vs. LCR-100, these six and a further nine genes (*Col3a1*, *Fbn1*, *Scx*, *Mkx*, *Bmp1*, *Tgfb1*, *Dcn*, *Tnc*, *Esr1*) were down-regulated. In addition, the repression of *Col3a1*, *Eln*, *Fbn1*, *Egr1* and *Tnc* was very strong in LCR-100; by more than 60% in comparison to HCR-100. 

Collagen type 3 is the “repair-collagen” [35], and ELN and FBN1 are the major components of the elastic fibers. The tenocyte markers MKX [36], SCX [37] and EGR1 are crucial transcription factors associated with tendon development and repair, may alter homeostasis and affect the maintenance and remodeling of tendons by regulating the expression of downstream tendon-related genes and matrix molecule genes. The procollagen C-proteinase BMP1 cleaves the C-terminus of procollagen I, II and III and induces accumulation of extracellular matrix [38]. TGFB1 has important and various effects on the metabolism of ECM components in tendons [39,40]. Additionally, *Dcn* and *Tnc* were repressed; both have essential roles in tissue homeostasis and function. DCN is necessary for maintaining collagen fibril structure and fiber [41,42,43]. TNC is a non-adhesive glycoprotein that has been described as a major player in the adaptation of tendon cells to mechanical loadings [44] and is important for the establishment and maintenance of the fibrocartilagenous regions of tendons [45]. Finally, expression of *Esr1* was repressed in LCR-100 in comparison to HCR-100 and its down-regulation may reflect the metabolic syndrome in LCR-100. It was shown that whole-body deletion of ESR1 in mice affects multiple tissues resulting in hyperinsulinemia, insulin resistance [46,47], impaired oxidative metabolism [48], increased inflammation [48], impaired glucose tolerance [46,47,49,50] and decreased physical activity [51] as well as increased fat and body mass [48,52,53]. Thus, ubiquitous knockout of *Esr1* results in a phenotype similar to that of metabolic syndrome [48,54,55].

Low intrinsic fitness resulted in a (partially strong) down-regulation of many genes in RC tendons, which are important for tendon development, repair and function, maintenance and remodeling.

These results indicate low intrinsic fitness:

- influences the RC tendons at least as much as age;
- may result in pronounced changes in the expression of ECM proteins, tendon-related genes and transcription factors that are essential for tendon formation, maturation, maintenance, remodeling, repair and function;
- affects especially aged tendons (the tendons of old LCR rats were more affected than the tendons of young LCR);
- may accelerate unhealthy aging with the pathogenesis of tendinopathy via repression of gene expression.

In a 2021 published study, the histological and molecular alteration in the Achilles tendon of this rat model were investigated [56]. An age-dependent effect was seen with down-regulation of ECM and inflammatory factors in the old rats. However, differences between the HCR and LCR rats were not detected in the Achilles tendon. This contrasts with the present study, which showed strain-dependent differences in the rotator cuff tendons. An explanation could be the functional and anatomical differences of the investigated tendons.

### 4.3. Limitations

The results of the presented study showed significant effects of aging and low intrinsic fitness on the histological appearance of the rotator cuff tendon and the expression of genes related to the ECM and tendon markers. The changes could affect the mechanical properties of the tissue and therefore analysis of the mechanical characteristics would have been interesting. To meet the 3R principles for more ethical animal use, we obtained the rotator cuff tissue from animals used in another experiment that did not affect tendon tissue. However, the number of animals available was therefore restricted and we had to prioritize the methods used to analyze the tissue. Therefore, no tissue was available for the mechanical testing of tendon properties.

## 5. Conclusions

The present findings demonstrate that a reduced cellularity and RNA content, reduction of the wavy pattern of collagen and elastic fibers, as well as reduced expression of genes related to ECM and tenocytes and the reduction of the *Col1*:*Col3* and *Col1*:*Eln* ratio, are associated with the aging of female RC tendons. Those features indicate a reduced metabolic activity and decreased tendon function and could be the reason for tendinopathies. 

Low intrinsic fitness additionally leads to increased RC tendon weight, enlarged tenocyte nuclei (consistent with degenerative tendons), and lower expression of genes important for tendon tissue. Intrinsic fitness affects the RC tendons at least as much as aging, probably more, as demonstrated by more regulated genes. The results indicate a faster aging of the tendons of LCR rats with a more pronounced predisposition for degenerative diseases. 

Some of the low intrinsic fitness-dependent features appear already in the young LCR: increased weight of the RC tendons, repressed expression of *Fn1*, *Eln*, *Mkx* and rise of the *Col1*:*Eln* ratio. 

Thus, low intrinsic fitness not only accelerates aging but may also be an independent risk for developing tendon diseases. 

## Figures and Tables

**Table 1:** qRT-PCR primer.

Gene	Accession Number	Primer Sequence	Amplicon Size (bp)
*18S rRNA*	NM_213557.1	forward: 5′tgtggtgttgaggaaagcag3′ reverse: 5′cctctatgggctcggatttt3′	240
*Ar*	NM_012502.1	forward: 5′tatcccagtcccagttgtgtta3′ reverse: 5′ccacagatcaggcaggtcttc3′	152
*Bgn*	NM_017087.1	forward: 5′gattgagaatgggagcctga3′ reverse: 5′ccttggtgatgttgttggag3′	143
*Bmp1*	NM_031323.1	forward: 5′caattaccccgacgattacc3′ reverse: 5′tacccacaatatcgcccaat3′	189
*Col1a1*	NM_053304.1	forward: 5′tgactggaagagcggagagt3′ reverse: 5′gatagcgacatcggcaggat3′	250
*Col3a1*	NM_032085.1	forward: 5′tgggatccaatgagggaga3′ reverse: 5′tcatggccttgcgtgttt3′	135
*Dcn*	NM_024129.1	forward: 5′gcagggaatgaagggtctc3′ reverse: 5′tccacaacggtgatgctatt3′	195
*Egr1*	NM_012551	forward:5′cacctgaccacagagtcctttt3′ reverse: 5′aaagtgttgccactgttggg3′	152
*Eln*	NM_012722.1	forward: 5′gtgtcggtcttccaggtgta3′ reverse: 5′gaaccttggccttgactcct3′	117
*Esr1*	NM_012689.1	forward: 5′gccttctacaggtccaattctga3′ reverse: 5′acagcacagtagcgagtctcc3′	119
*Fbn1*	NM_031825.1	forward: 5′gtgtgaactgagcgcgaac3′ reverse: 5′cactggccaccatcacagata3′	288
*Fn1*	NM_019143.2	forward: 5′tcccacgatccgatgatgt3′ reverse: 5′tccacacggtatccagtcac3′	118
*Mkx*	XM_017600733.1	forward: 5′gctctaggctcgcagatgac3′ reverse: 5′gcgttgccctgaacatactt3′	143
*Postn*	NM_001108550	forward: 5′tagggtgtgagggagacagc3′ reverse: 5′caggtccgtgaaagtggttt3′	170
*Scx*	NM_001130508.1	Qiagen (QT01596028)	
*Tgfb1*	NM_021578.2	forward: 5′aactgtggagcaacacgtagaa3′ reverse: 5′tattccgtctccttggttcag3′	157
*Tgfb3*	NM_013174.2	forward: 5′gagggtggaagccattagg3′ reverse: 5′gcagactgccagttcattgtg3′	256
*Tnc*	NM_053861.1	forward: 5′atgttccaaagagccagcaa3′ reverse: 5′aggctgtagttgaggcggta3′	247
*Tnmd*	NM_022290.1	forward:5′ggcccgaggtatccaagaag3′ reverse: 5′agatgccagtgtatccgttttt3′	177

**Table 2:** Cell number and nucleus area of HCR and LCR.

Group	HCR-10	HCR-100	LCR-10	LCR-100
Cell number	149 (113–189)	60 (43–61) ^a^	99 (79–118)	47 (39–56) ^b^
Area nucleus µm^2^	30.9 (28.5–32.8)	28.4 (27.8–29.5)	37.8 (36.7–38.5)	33.3 (31.8–34.8) ^c^

^a^ and ^b^ indicate significant differences in cell number in comparison to young animals of the same strain; ^c^ indicates significant differences to HCR-100. Data are given as median and interquartile range (Q1–Q3), n = 7 per group.

## Data Availability

The original data presented in the study are included in the article and queries can be directed to the corresponding author.

## Animal Model Availability

The LCR-HCR rats are maintained as an animal model resource at The University of Toledo, Toledo, OH. Contact LGK (Lauren.Koch2@UToledo.Edu) or SLB (brittons@umich.edu) for information.
